# Nonbacterial thrombotic endocarditis in antiphospholipid syndrome, presenting with severe mitral stenosis, heart failure, and stroke: case report

**DOI:** 10.1093/ehjcr/ytag409

**Published:** 2026-06-03

**Authors:** Hussein Abd AlMejbel, Munthir Abdullah AlAhmed, Abdulrahman Musaad Alharbi, Samah Matuq Ali, Murouj Jamil Alansari

**Affiliations:** Department of Cardiology, Security Forces Hospital Makkah, 24251, KSA; Department of Internal Medicine, Security Forces Hospital Makkah, 24251, KSA; Department of Internal Medicine, Security Forces Hospital Makkah, 24251, KSA; Department of Cardiology, Security Forces Hospital Makkah, 24251, KSA; Department of Cardiology, Security Forces Hospital Makkah, 24251, KSA

**Keywords:** Nonbacterial Thrombotic Endocarditis, Antiphospholipid Syndrome, Severe Mitral Stenosis, Stroke, Case report

## Abstract

**Background:**

Nonbacterial thrombotic endocarditis (NBTE) is a rare cardiac manifestation of antiphospholipid syndrome (APS) and may also occur in patients with malignancy. It can mimic infective endocarditis or rheumatic mitral valve disease and often results in valvular disease and embolic complications.

**Case summary:**

A 39-year-old man with a 10-year history of primary APS presented with acute decompensated heart failure and left-sided weakness. Clinical examination revealed signs of biventricular failure, mid-diastolic and systolic murmurs, and neurological deficits. Transthoracic echocardiography (TTE) revealed severe mitral stenosis, large mitral valve masses, severe tricuspid regurgitation, and pulmonary hypertension. Brain magnetic resonance imaging (MRI) showed a lacunar infarct. Three sets of blood cultures were negative. Transoesophageal echocardiography (TEE) revealed large mobile masses on the mitral valve leaflets. The differential diagnosis included infective endocarditis and NBTE. In the setting of APS and the absence of fever, along with persistently negative blood cultures, NBTE was strongly suspected. Given the presence of severe obstructive mitral stenosis and a life expectancy exceeding one year, the management plan included valvular intervention in collaboration with a multidisciplinary team, alongside supportive therapy and anticoagulation. However, the patient declined surgical intervention and was therefore managed conservatively with optimized medical therapy and anticoagulation. On follow-up, the patient demonstrated clinical improvement with corresponding improvement in echocardiographic parameters.

**Discussion:**

NBTE should be considered in APS patients presenting with cardiac murmurs and embolic events. TTE is essential for diagnosis. This case underscores the importance of prompt identification and a multidisciplinary approach.

Learning points

- Nonbacterial thrombotic endocarditis is a rare but severe valvular manifestation of antiphospholipid syndrome.
- Transoesophageal Echocardiography is essential for diagnosis when Nonbacterial thrombotic endocarditis is suspected.
- antiphospholipid syndrome patients who exhibit symptoms of embolic stroke and heart failure should be assessed for Nonbacterial thrombotic endocarditis.

## Introduction

APS is a systemic autoimmune disorder associated with both arterial and venous thrombotic events, as well as abnormalities in cardiac valves. NBTE, although rare, represents a serious cardiac complication characterized by the deposition of sterile platelet thrombi on heart valves, especially the mitral and aortic valves. In this report, we present a case of NBTE in a patient with long-term primary APS who experienced heart failure due to mitral stenosis (mechanical obstruction) and a cerebral embolic stroke.

## Summary figure

**Figure:**
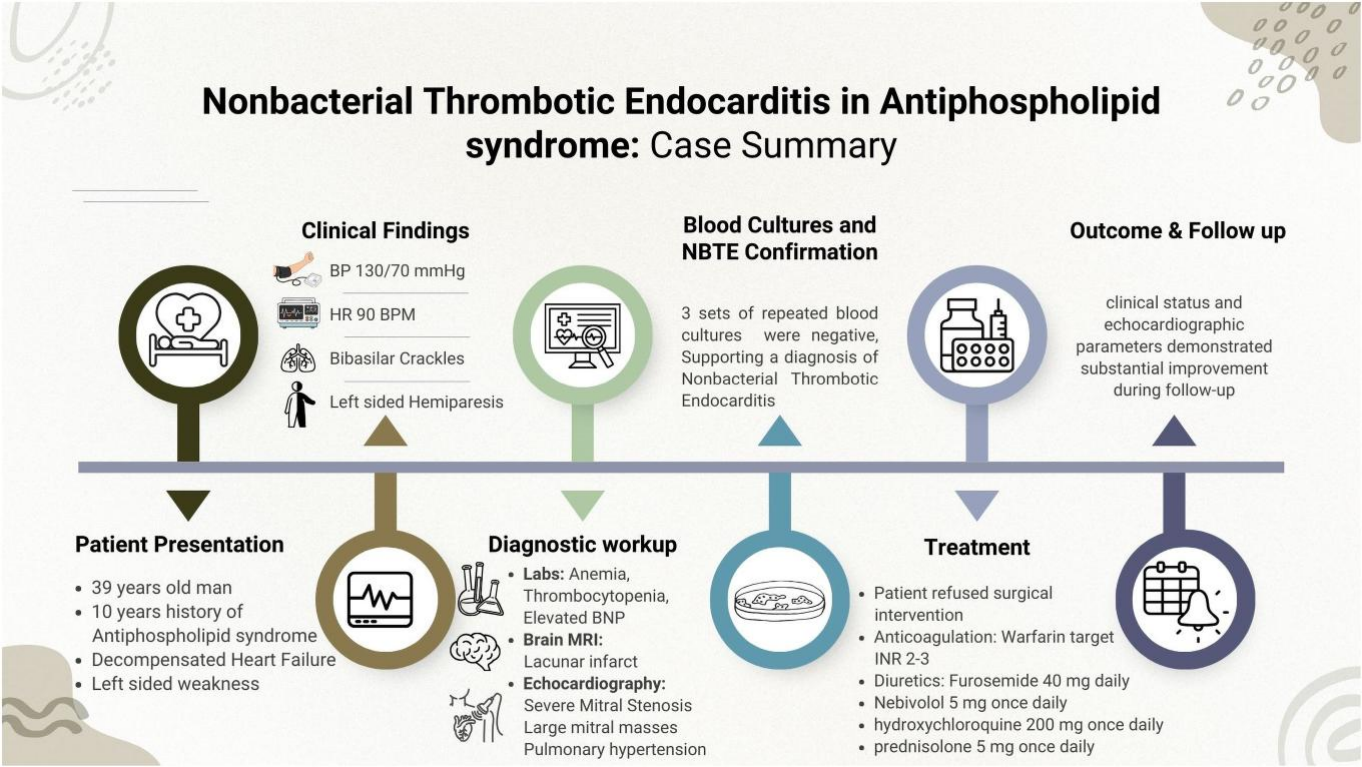


## Case presentation

A 39-year-old man with a 10-year history of Primary Antiphospholipid Syndrome (PAPS), diagnosed following multiple thrombotic events, along with ischaemic heart disease, epilepsy, and chronic kidney disease. The patient had been prescribed long-term warfarin therapy for primary antiphospholipid syndrome; however, he was not compliant with regular follow-up in the anticoagulation clinic. His last documented visit, three months before presentation, which showed an International Normalized Ratio (INR) of 1.40, indicating subtherapeutic anticoagulation. The patient arrived at the emergency room (ER) with complaints of exertional dyspnoea, orthopnoea, and left-sided weakness that began approximately 10 h before admission. Upon examination, the patient was conscious and alert but in respiratory distress. Vital signs included blood pressure of 130/70 mm Hg, heart rate of 90 bpm, respiratory rate of 20 breaths per minute, and oxygen saturation of 90% on room air. Physical examination revealed elevated jugular venous pressure (JVP) with significant V waves, bilateral air entry with bibasal crepitations, and cardiac auscultation that indicated loud S2, mid-diastolic murmur at the apex, and systolic murmur at the left lower sternal border. Neurological examination revealed left-sided weakness in both upper and lower limbs, with power rated at 3/5 and bilateral lower limb oedema. Based on the initial assessment, mitral stenosis complicated by pulmonary hypertension, which resulted in symptoms of heart failure, was suspected.

Initial laboratory investigations revealed the following: Haemoglobin 9 g/dL (reference range: 13.5–17.2 g/dL), platelets 130 000/µL (150 000–450 000/µL), white blood cell count 9000/µL (4000–11 000/µL), troponin 0.01 ng/mL (0–0.026 ng/mL), brain natriuretic peptide 1500 pg/mL (0–160 pg/mL), erythrocyte sedimentation rate 80 mm/Hr (0–14 mm/Hr), alanine aminotransferase 74.0 U/L (0–55 U/L), alkaline phosphatase 188 U/L (40–150 U/L), aspartate aminotransferase 75 U/L (5–34 U/L), total bilirubin 40 µmol/L (3.4–20.5 µmol/L), direct bilirubin 29 umol/L (0–9 µmol/L), and INR 1.71 (0.89–1.1). Electrocardiography revealed a normal sinus rhythm with left and right atrial enlargement and signs of right ventricular strain. Chest X-ray showed cardiomegaly and pulmonary congestion. A CT scan of the brain was unremarkable. Intravenous furosemide was initiated, resulting in clinical improvement of dyspnoea.

**Figure 1:**
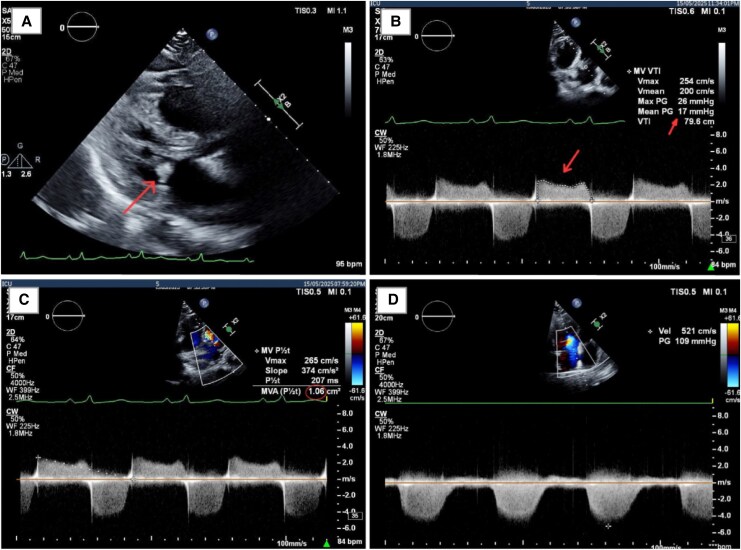
Transthoracic echocardiography; (*A)* parasternal long axis view showing a large mass on mitral valve leaflets. (*B*) Apical 4 chamber view with CW on mitral valve, Mean gradient 17 mmHg (severe mitral stenosis), and MR +2. (*C*) Apical 4 Chambers view, with CW on MV. P1/2t 207 ms, and mitral valve area (MVA) 1.06 cm^2^. (*D*) Apical 4 chambers view with CW on Tricuspid valve, severe tricuspid regurgitation (TR), a systolic pulmonary artery pressure (SPAP) exceeding >110 mmHg.

**Figure 2:**
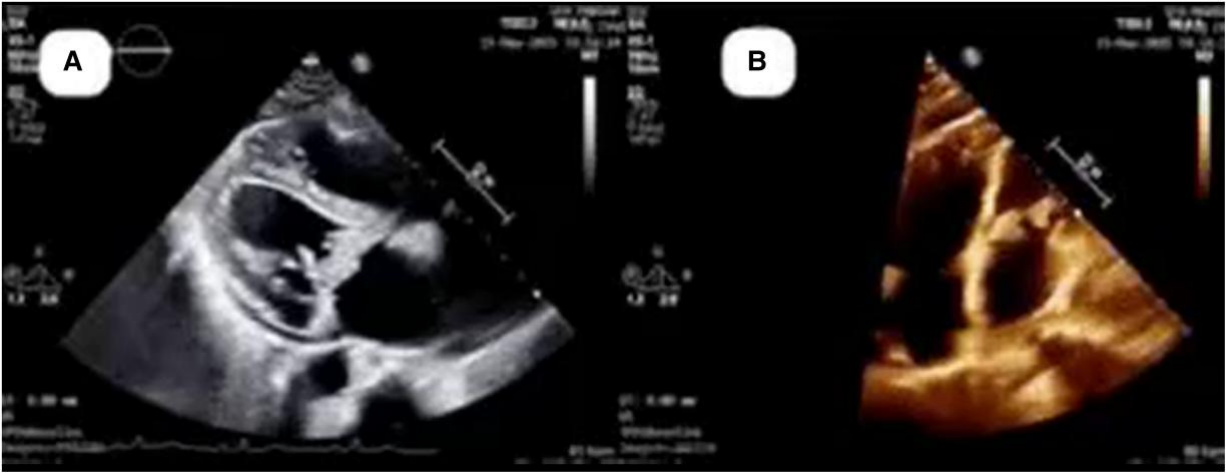
Transthoracic echocardiography; (GIF *A*): parasternal long axis view, showing large masses causing mitral valve obstruction. (GIF *B*): Apical 4 chambers view showing right ventricular dilatation (42 mm at the base) and dysfunction, and biatrial enlargement.

Transthoracic echocardiography performed in the emergency department demonstrated large mobile masses attached to both mitral valve leaflets, resulting in severe mitral stenosis. Left ventricular systolic function was preserved (ejection fraction 50%). The right ventricle was dilated (basal diameter 4.57 cm) with reduced systolic function (TAPSE 14.1 mm, S′ 7.7 cm/s). Severe tricuspid regurgitation was present, along with biatrial enlargement. No pericardial effusion or additional significant valvular abnormalities were identified. (*Figures 1* and *2*).

Differential diagnoses included infective endocarditis (IE) and nonbacterial thrombotic endocarditis (NBTE). Given the lack of fever and the patient’s history of PAPS, NBTE was deemed more likely pending blood culture results.

**Figure 3:**
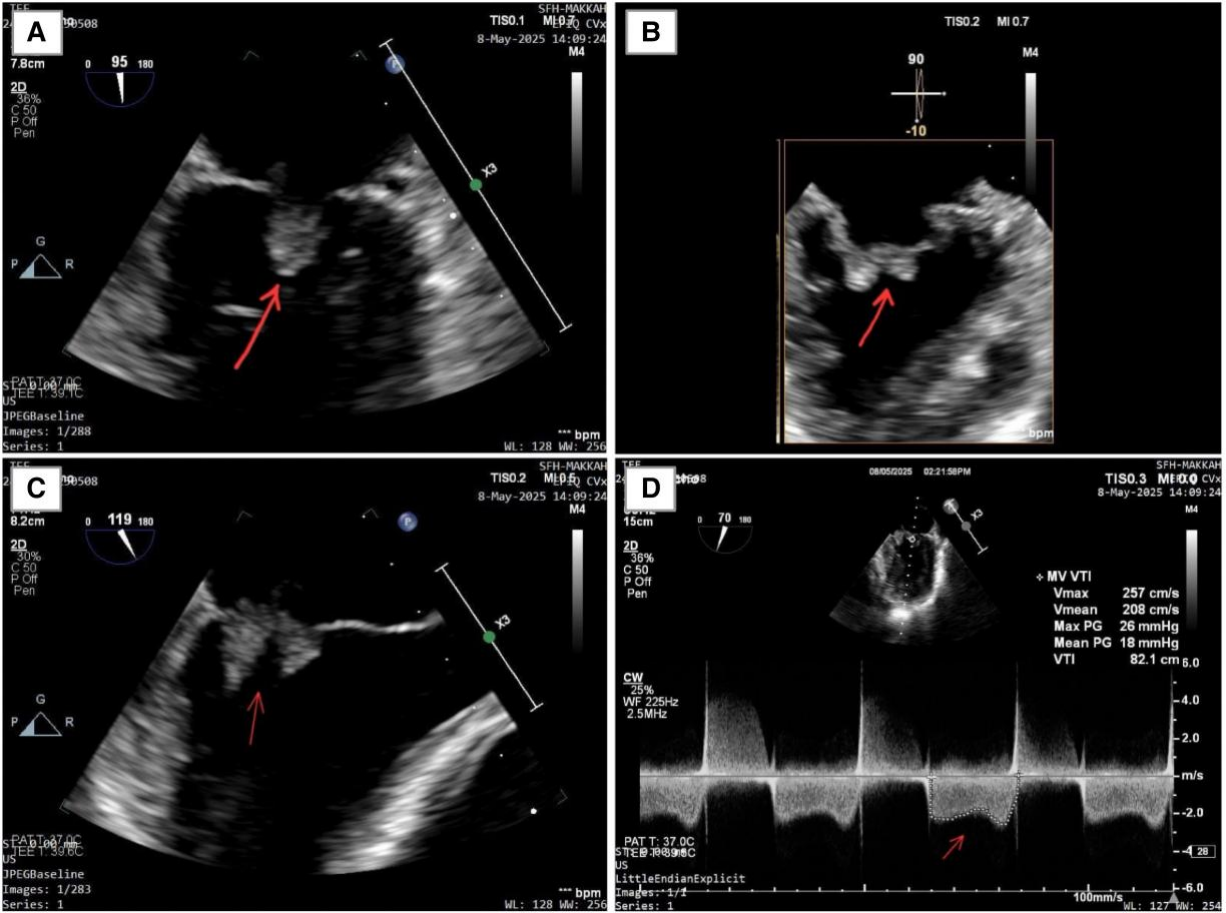
Transoesophageal echocardiography revealed; (*A*) and (*B*) 2 chambers view revealing large mitral valve masses attached to the tips of the mitral leaflets and on the A1/P1 segments, measuring approximately 11–12 mm by 13–15 mm. These masses giving a ‘kissing’ appearance. (*C*) TEE Long axis 3 champers view. (*D*) 2 chambers view with a Continuous Wave (CW) Doppler on mitral valve revealing mean gradient 18 mmHg (severe mitral stenosis), and Mitral regurgitation +2.

**Figure 4:**
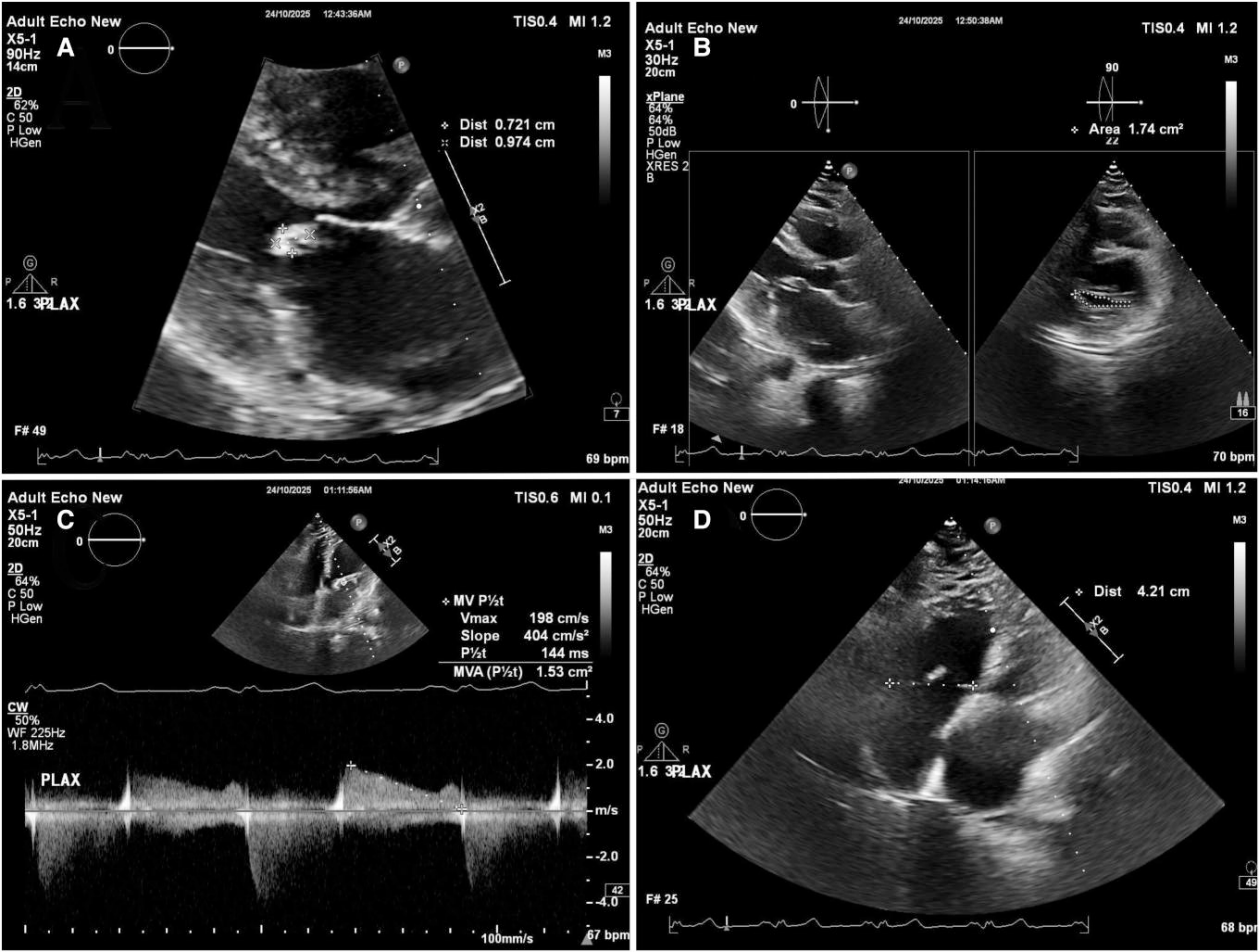
Follow-up transthoracic echocardiography (6-month follow-up). (*A*) Parasternal long-axis view demonstrating marked reduction in mitral valve mass size, measuring approximately 0.72 × 0.97 cm. (*B*) Short-axis view of the mitral valve with direct planimetry showing a mitral valve area of 1.74 cm^2^. (*C*) Continuous-wave Doppler across the mitral valve demonstrating pressure half-time (P1/2t) of 144 ms with calculated mitral valve area (MVA) of 1.53 cm^2^. (*D*) Apical four-chamber view showing right ventricular basal diameter measuring 4.21 cm, indicating improvement in right ventricular dilatation.

Subsequent MRI of the brain identified an acute lacunar infarct (*Figure 3*). A transoesophageal echocardiogram (TEE) was performed for further evaluation, which revealed large masses resulting in mechanical obstruction across the valve orifice. Notably, there was no evidence of valve destruction observed (*Figure 4*). Three sets of blood cultures returned negative, further supporting the diagnosis of NBTE. Screening for malignancies, including CT of the abdomen and pelvis and PSA testing, yielded negative results.

The patient was ultimately diagnosed with nonbacterial thrombotic endocarditis complicated by severe mitral stenosis, heart failure, and stroke. Following multidisciplinary team discussion involving cardiology, cardiac surgery, neurology, and rheumatology, mitral valve intervention was recommended due to severe obstructive mitral stenosis and prior embolic stroke. However, the patient and his family declined surgical intervention after detailed counselling regarding risks and benefits. He was therefore discharged on optimized medical therapy, including oral furosemide 40 mg once daily, prednisolone 5 mg once daily, nebivolol 5 mg once daily, hydroxychloroquine 200 mg once daily, and warfarin 5 mg once daily with a target INR of 2.0–3.0. He was followed regularly in the anticoagulation clinic as well as cardiology and rheumatology outpatient clinics. The last recorded INR before discharge was 2.8, consistent with therapeutic anticoagulation.

At the 6-month follow-up, the patient demonstrated significant clinical and echocardiographic improvement under conservative management after declining surgery. Functional status improved from NYHA class III–IV at presentation to NYHA class II, with complete resolution of orthopnoea and paroxysmal nocturnal dyspnoea. Diuretic requirement decreased substantially, and the patient was gradually weaned off regular furosemide, currently using it on an as-needed basis only. Follow-up echocardiography showed a marked reduction in mitral valve mass size from approximately 11 mm × 15 mm to 0.72 cm × 0.97 cm. The mitral valve area improved from 1.06 cm^2^ to 1.53 cm^2^ by pressure half-time and 1.7 cm^2^ by direct planimetry, indicating partial relief of obstruction. Mitral regurgitation improved from moderate (+2) to mild (+1), and tricuspid regurgitation decreased from severe to mild (+1). Severe pulmonary hypertension (previously >110 mmHg) significantly declined to 61 mm Hg. Right ventricular basal diameter decreased from 4.57 to 4.2 cm, with recovery of systolic function, as reflected by improvement in TAPSE from 14.1 mm to 1.78 cm and S′ velocity from 7.7 to >10 cm/s. The inferior vena cava normalized in size with preserved respiratory collapse. Both clinical status and echocardiographic parameters showed marked improvement during follow-up.

## Discussion

Antiphospholipid syndrome (APS) is an autoimmune prothrombotic disorder that leads to both arterial and venous thrombosis. A notable yet rare manifestation of APS is nonbacterial thrombotic endocarditis (NBTE), which has a reported prevalence of 0.3% to 9.3% in autopsy studies.^1^ NBTE is characterized by sterile masses on cardiac valves, predominantly affecting the mitral and aortic valves, with sizes that can vary significantly. While malignancies are commonly associated with NBTE, accounting for up to 80% of cases, it can also occur in autoimmune conditions such as systemic lupus erythematosus and primary APS.^2,3^ Patients with NBTE may often be asymptomatic; however, they can present with clinical findings such as valvular masses, systemic embolism, or, in rare instances, acute heart failure. Unlike infective endocarditis (IE), NBTE usually does not lead to valve destruction, although it can rarely result in significant valvular dysfunction.^4–6^ On the other hand, in rare cases NBTE can present as Congestive Heart Failure.^7^ Diagnosis of NBTE requires the identification of valvular masses via echocardiography and the exclusion of infective endocarditis, with Blood cultures should be repeatedly negative. Transoesophageal echocardiography (TEE) is the preferred diagnostic tool, offering a sensitivity of up to 90%.^8^

In our case, large masses on the mitral valve leaflets caused severe mitral stenosis (MS), which resulted in heart failure and stroke. The patient had a background of primary APS, with no signs of infection confirmed through persistently negative blood cultures and a thorough malignancy screening. These findings strongly support a diagnosis of NBTE. In APS, valve disease is characterized by the existence of valve masses and/or moderate to severe dysfunction, in the absence of rheumatic fever or infective endocarditis.^9^ Valvular heart disease is a recognized cardiac manifestation of antiphospholipid syndrome (APS). According to the revised Sapporo (Sydney) classification criteria (2006), valve involvement is considered a common non-criteria manifestation rather than a formal diagnostic criterion.^10^ In contrast, the 2023 ACR/EULAR updated APS classification criteria newly incorporate cardiac valve disease as a weighted clinical domain, defined by echocardiographic evidence of valve thickening or vegetations in the absence of infection.^11^ This inclusion highlights the clinical importance of valvular involvement in APS.

NBTE differs substantially from infective endocarditis (IE) in both management and prognosis. NBTE is managed primarily with therapeutic Anticoagulation due to their high risk of systemic embolization. The preferred anticoagulants in APS-related NBTE are either unfractionated heparin or low-molecular-weight heparin, followed by long-term warfarin with a target INR of 2.0–3.0.^8,9^ Surgical intervention may be warranted for patients with severe valvular dysfunction, recurrent thromboembolic events despite adequate anticoagulation, or when long-term survival is anticipated to provide meaningful benefit.^6,8^ There is no role for antimicrobial therapy, and treatment focuses on the underlying prothrombotic condition, such as malignancy or autoimmune disease.^4,8,12^ The role of corticosteroids and other immunosuppressive therapies in NBTE related to APS remains uncertain, although some case reports suggest that steroid therapy combined with anticoagulation and hydroxychloroquine may promote regression of masses.^9^ Further research is needed to clarify their efficacy. In contrast, IE requires prolonged targeted intravenous antibiotics based on blood culture results, typically for 4–6 weeks, and surgery is indicated for heart failure, persistent infection, or large vegetations (>15 mm).^8^ Antithrombotic therapy is generally not indicated in IE except in specific comorbidities. Prognostically, NBTE outcomes are largely determined by the underlying disease, with one-year mortality rates of 33–38%, mostly due to cancer progression; embolic events are frequent, while valvular dysfunction is usually less severe.^4,8,12^ IE, while still associated with morbidity and mortality, has a more favourable prognosis with one-year survival around 85–90%, and adverse outcomes are influenced by heart failure, age, comorbidities, and failure to perform indicated surgery.^13^

**Table 1:** Comparison between NBTE and IE

Criteria	NBTE	Infective endocarditis (IE)
Clinical Presentation	Often **asymptomatic**; may present with **systemic emboli**	Fever, Chills, heart murmurs, Fatigue, sepsis, emboli phenomenon.
Underlying condition	Common in patients with **APS**, Malignancies, or hypercoagulable states	Common in patients with valvular disease, Intravenous drug use, or prosthetic valves
Histopathology	Thrombotic mass, No infection	Infective vegetation with bacterial colonies
Blood Cultures	Negative	Positive for bacteria
Imaging findings	Echocardiography shows masses, may form ‘**kissing lesions**’ on opposing leaflets No signs of infection ( e.g. abscess) Rarely valve destruction	Echocardiography shows vegetations Abscess **Valve destruction:** perforation, flail leaflet. Regurgitation
Treatment	Treat underlying cause Anticoagulation therapy Surgery in advanced mechanical obstruction	Antibiotic therapy Surgical therapy when indicated for e.g : (1) Large vegetation >15 mm (2) Abscess (3) Persistent infection (4) Heat failure due to valvular dysfunction (5) Recurrent embolic events with persistent large vegetation ≥10 mm
Prognosis	Depends on the underlying cause	Risk of complications high if untreated; prognosis better with early therapy

In patients with NBTE, the decision between conservative and surgical management depends on the severity of valvular involvement, the risk of embolization, and the patient’s overall prognosis. Anticoagulation remains the cornerstone of therapy. However, in our case, mitral valve replacement was indicated due to significant valvular obstruction and recurrent embolic episodes despite adequate anticoagulation. Decisions regarding the surgical management of NBTE should involve a multidisciplinary team, including cardiology, cardiac surgery, neurology, and rheumatology specialists.

## Supplementary Material

ytag409_Supplementary_Data

## Data Availability

The data underlying this article will be shared on reasonable request to the corresponding author.

## Lead author biography

Dr. Hussein Almejbel is a Cardiologist at security forces hospital Makkah. Syrian board-certified in Cardiology. Arab board-certified in Cardiology. FACC, EACVI, NBE, HF certified.

## Notes

**Consent:** We have received the patient’s informed consent for the use of data from their medical records and clinical images obtained for publication, in accordance with COPE guidelines.
